# Size Effects of Silver Nanoparticles and Magnetic Beads on Silver-Gold Galvanic Exchange in Aptamer-Based Electrochemical Assays

**DOI:** 10.3390/bios15120768

**Published:** 2025-11-25

**Authors:** Eman Alwarsh, Trang Bui, Marco Cardenas, Daniel Adrian, Charuksha Walgama

**Affiliations:** Department of Mathematical, Applied, and Physical Sciences, University of Houston-Clear Lake, 2700 Bay Area Boulevard, Houston, TX 77058, USA

**Keywords:** silver nanoparticles, magnetic conjugates, galvanic exchange, particle size, electrochemical aptamer sensors

## Abstract

In this work, we investigated the influence of silver nanoparticle (AgNP) size (diameters of 20, 50, and 100 nm) and magnetic bead (MB) size (diameters from 100 to 4500 nm) on silver-gold galvanic exchange signal generation in magnetic electrochemical assays. Two conjugation strategies, including biotin-streptavidin interaction and a streptavidin-specific aptamer interaction, were compared to assess differences in binding chemistry and conjugation efficiency. Calibration studies showed that 50 nm diameter AgNPs provided the best sensitivity and galvanic exchange efficiency, yielding the lowest detection limits across both conjugation strategies. Larger AgNPs produced stronger signals but reached saturation rapidly, whereas smaller particles required higher concentrations to achieve equivalent silver content. Among MBs, 1000 nm beads consistently gave the highest galvanic exchange efficiency, offering sufficient surface area for AgNP loading while minimizing steric hindrance and electrode obstruction. These findings were confirmed by complementary electrochemical impedance spectroscopy, UV-Vis absorbance, and SEM imaging, which collectively demonstrated the strong influence of bead size on charge transfer resistance and conjugation efficiency. Overall, the combination of 50 nm AgNPs with 1000 nm MBs emerged as the optimal configuration, providing improved sensitivity and reproducibility. We believe these results offer valuable design guidelines for the development of next-generation aptamer-based electrochemical biosensors for biomarker detection.

## 1. Introduction

In recent years, aptamer-based electrochemical biosensors have gained considerable attention due to their enhanced sensitivity for biomarker detection [1]. These assays replace antibodies by aptamers, which provide high affinity and specificity, and combine them with electrochemical techniques to generate amplified analytical signals through coupled redox reactions.

Although antibodies traditionally offer high selectivity and binding affinity to target molecules, their production is often time-consuming, costly, and complex [2]. In contrast, aptamers, which are single-strand nucleic acid sequences can be synthesized within a short time and readily modified with desired functional groups [3,4]. Consequently, aptamers have emerged as attractive alternatives to antibodies for biosensing applications.

**Scheme 1:**
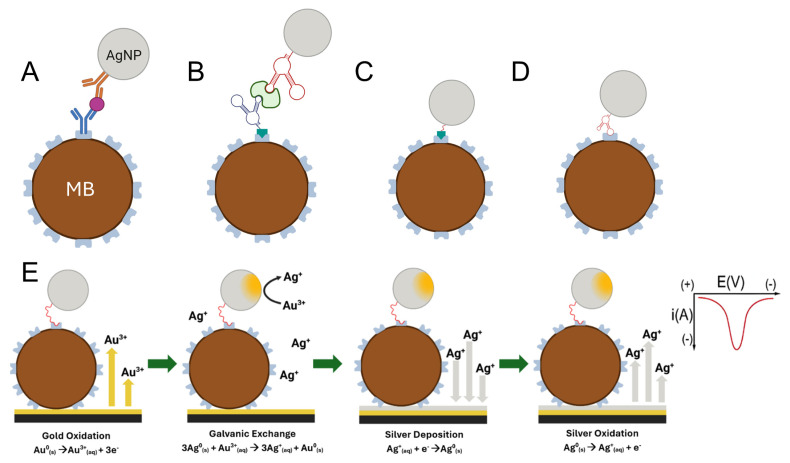
Schematic representation of various strategies for conjugating silver nanoparticles to streptavidin-coated magnetic beads. (**A**) Formation of a traditional dual-antibody immunocomplex. (**B**) Assembly of a dual-aptamer-based immunocomplex. (**C**) Model assay based on biotin-streptavidin-mediated conjugation. (**D**) Model assay utilizing a streptavidin-specific aptamer for AgNP conjugation. (**E**) Silver-gold galvanic exchange process for electrochemical detection.

This study specifically examines a magnetic electrochemical assay, where magnetic beads (MBs) serve as analyte-concentrating agents and silver nanoparticles (AgNPs) function as electrochemically active labels to generate analytical signals [5]. Scheme 1 illustrates typical assay configurations: dual-antibody-based immunocomplexes (Scheme 1A) and dual-aptamer-based immunocomplexes (Scheme 1B). For practical evaluation, we investigate two model assays: one in which AgNPs are directly conjugated to streptavidin-coated magnetic beads via biotin-streptavidin interactions (Scheme 1C), and another in which AgNPs are conjugated via a streptavidin-specific aptamer (Scheme 1D). These model assay formats were used to study the size-dependent galvanic exchange behavior independently of biological binding effects.

Nanomaterial-based electrochemical sensors have gained prominence for their ability to amplify electron transfer and enhance analytical sensitivity through nanoscale surface interactions [6,7,8]. Recent studies have demonstrated that AgNPs are highly effective electrochemical labels due to their sharp stripping voltammetric signals and high charge capacity, which can significantly enhance detection sensitivity in biosensing applications [5]. In particular, the silver-gold galvanic exchange reaction has emerged as a powerful mechanism for converting target-binding events into quantifiable electrochemical signals [9,10,11]. This reaction is initiated when AgNPs, conjugated to a biorecognition element, come into contact with electrogenerated Au^3+^ ions. The more noble gold ions spontaneously oxidize the zero-valent silver atoms in the nanoparticles, forming a gold shell while releasing Ag^+^ ions. The released silver ions are then electrochemically deposited and quantified via anodic stripping voltammetry (ASV), providing a signal that is directly proportional to the analyte concentration in a sandwich-type assay (See Scheme 1E) [12].

This galvanic exchange mechanism has been successfully incorporated into magnetic bead-based immunoassays, where AgNP-labeled MBs enable preconcentration of the analyte and enhance detection sensitivity [9,13]. The Crooks group recently introduced a paper-based immunosensor for detecting heart failure biomarker: NT-proBNP using AgNP-labeled MBs and galvanic exchange-based electrochemical readout. Their subsequent work further demonstrated that assay performance can be tuned by manipulating the shape and size of the AgNPs, which impacts galvanic exchange efficiency and signal transduction [12,14,15,16,17,18].

The size of both AgNPs and MBs plays a pivotal role in determining the sensitivity and efficiency of electrochemical immunoassays. Prior studies have shown that smaller AgNPs exhibit faster oxidation kinetics and enhanced surface reactivity, whereas larger particles provide higher overall charge but may suffer from limited galvanic exchange efficiency due to incomplete core oxidation and passivation by a gold shell [19] Interestingly, reducing the size of gold nanoparticles has also been shown to improve the thermodynamics of galvanic exchange reactions, favoring more complete silver oxidation [20]. The size and shape of AgNPs also influence their optical and electrochemical properties, further affecting signal generation [21,22]. Similarly, MB size significantly impacts the binding efficiency, steric accessibility, and magnetic responsiveness of the assay. Smaller MBs offer higher surface area-to-volume ratios but may be more difficult to manipulate magnetically, while larger MBs facilitate more efficient preconcentration but may limit accessibility of biorecognition elements due to steric hindrance [23,24]. Taken together, these findings underline the importance of optimizing both AgNP and MB sizes to maximize electrochemical signal output and assay performance.

Despite these advances, limited studies have systematically examined how AgNP diameter and MB size influence the efficiency of silver-gold galvanic exchange and the resulting electrochemical response. To address these knowledge gaps, this study systematically investigates the effects of AgNP size (diameters of 20, 50, and 100 nm) and magnetic bead size (diameters of 100, 200, 1000, 3000, and 4500 nm) on the electrochemical signal output in two model aptamer-based magnetic immunoassays utilizing silver-gold galvanic exchange detection. Therefore, in this study we investigate how AgNP size influences the density of nanoparticle labeling on magnetic beads, the steric accessibility of the recognition layer, and the resulting assay sensitivity and reproducibility. By systematically examining these factors, our work provides new insights into the relationship between nanoparticle size, conjugation behavior, and electrochemical signal generation, offering design guidelines for more reliable and sensitive biosensing platforms.

## 2. Experimental Section

### 2.1. Chemicals and Materials

All solutions were prepared using Milli-Q ultrapure water (resistivity 18.0 MΩ·cm). Chemical reagents for HEPES buffer (100 mM, pH 7.4), borate buffer (100 mM, pH 7.5), citrate buffer (100 mM, pH 3; a mixture of sodium citrate dihydrate and citric acid monohydrate), and BCl buffer (100 mM, pH 7.4; a mixture of borate buffer and KCl) were obtained from Sigma-Aldrich(St. Louis, MO, USA). Citrate-capped AgNPs with diameters of 20, 50, and 100 nm (0.02 mg/mL) were purchased from NanoComposix (San Diego, CA, USA). A 12-base biotinylated thiolated ssDNA (5′ACATTAAAATTC3′) and a 96-base thiolated streptavidin-specific DNA aptamer(5′ATACCAGCTTATTCAATTGATGCAGCCACCTACATTGCAGGTTTTATAATACAACTACGCGCCGTTCGCAGGCTAGTTAGATAGTAAGTGCAATCT3′) [25,26] were obtained from Biosearch Technologies (Petaluma, CA, USA) and diluted with Milli-Q water prior to use. Streptavidin-coated magnetic beads with nominal diameters of 100, 200, 1000, 3000, and 4500 nm were purchased from Ocean Nanotech (San Diego, CA, USA). Low-retention siliconized microcentrifuge tubes were sourced from Fisherbrand (Pittsburgh, PA, USA). Tetrachloroauric acid (HAuCl_4_, 6.0 mM in 0.10 M KNO_3_) was used for electrode modification. Conductive carbon paste (Cl-2042) was obtained from Nagase ChemteX America LLC (New York, NY, USA), and mesoporous printing media (8.5 × 11 in sheets) were obtained from Novacentrix (Austin, TX, USA). Magnetic separations were carried out using a neodymium block magnet.

### 2.2. Instrumentation

Electrochemical detection was performed on a CH Instruments (Austin, TX, USA) 650E electrochemical workstation. UV-vis measurements were conducted using a Thermo Scientific (Waltham, MA, USA) Evolution 60S UV-Visible Spectrophotometer. SEM images were obtained using a Thermo Scientific (Waltham, MA, USA) FEI Dual Beam 235 Focused Ion Beam system.

### 2.3. Fabrication of Screen-Printed Electrodes

A slight modification of a previously published procedure was used to fabricate the electrodes [13]. Briefly, electrode stencils were patterned onto an overhead transparency paper using a Cricut Maker 3 cutting machine. A stencil was then aligned over the hydrophilic surface of the plastic mesoporous printing media, and conductive carbon paste was applied to define the three electrode regions. The printed electrodes were air-dried overnight at ambient conditions. To confine the reaction area, a hydrophobic barrier was applied around the electrode using nail polish. Photographs of the screen-printed electrode fabrication process and the complete electrode assembly are provided in Figure S1 of the Supplementary Information.

The working electrode surface was modified by electrodepositing gold. A 50.0 µL droplet of 6.0 mM HAuCl_4_ in 0.10 M KNO_3_ was placed on the electrode, and the potential was stepped from 0.00 to −0.60 V for 2.0 s using the electrochemical workstation. The electrode was then rinsed twice with ultrapure water (18.0 MΩ·cm) and gently dried with a tissue.

### 2.4. Preparation of MB-AgNP Model Conjugates

The conjugation of 12-base or 96-base ssDNA to AgNPs was carried out using a previously reported pH-assisted method [13,27]. Briefly, 2.33 μL of 0.22 mM thiol-DNA-biotin or thiol-streptavidin aptamer was added to 400.0 μL of 0.02 mg/mL citrate-capped AgNPs in a 1.5 mL low-retention, siliconized microcentrifuge tube. The mixture was incubated at 23 °C for 5 min at 400 rpm in an orbital incubator shaker. To facilitate conjugation, 26.5 μL of citrate buffer (100 mM, pH 3) was added, and incubation was continued under the same conditions for 5 min. An additional 27.8 μL of citrate buffer was then added, followed by a 25 min incubation at 400 rpm.

Following conjugation, 400.0 μL of HEPES buffer (100 mM, pH 7.5) was added, and the solution was centrifuged at 16,600× *g* for 20 min at 20 °C. The supernatant was removed, and the pellet was resuspended in 400.0 μL of borate buffer (100 mM, pH 7.4). This washing step was repeated once, and the final pellet was resuspended in 400.0 μL of BCl buffer (100 mM, pH 7.4).

For immobilization of AgNP-DNA onto streptavidin-coated MBs, 16.0 μL of MB suspension was transferred to a separate low-retention microcentrifuge tube and washed twice by magnetic separation, discarding the supernatant and resuspending the pellet in 16.0 μL of BCl buffer. The washed MBs were mixed with 100.0 μL of AgNP-DNA conjugate and incubated at 1400 rpm for 30 min at 20 °C. The resulting AgNP-DNA-streptavidin-MB conjugates were washed twice with BCl buffer and resuspended in 16.0 μL of BCl buffer for storage at 4 °C. MB-AgNP conjugates were freshly prepared for each experiment.

### 2.5. Electrochemical Detection

Electrochemical detection was performed using ASV followed by the Ag-Au galvanic exchange process. The MB-AgNP conjugates (2.0 μL) were mixed with 48.0 μL of BCl buffer and deposited onto the gold-coated working electrode of the screen-printed electrode. The conjugates were preconcentrated on the working electrode surface using a magnet which was located under the working electrode on 3D printed plastic platform (Figure S1D). Chronoamperometry was then applied by stepping the potential from 0.00 to 0.80 V to oxidize Au^0^ to Au^3+^. The electrogenerated Au^3+^ initiated a GE reaction, oxidizing Ag to Ag^+^ while being reduced back to Au^0^. A subsequent CA step at −0.70 V reduced Ag^+^ to Ag^0^ on the electrode surface. This oxidation-reduction sequence was repeated twice, followed by a linear sweep voltammetry scan from −0.70 V to 0.20 V to oxidize deposited Ag^0^. The resulting oxidation current was recorded, and the total charge was determined by integration of the ASV trace.

Electrochemical impedance spectroscopy was performed using the same carbon-based screen-printed electrode system in a three-electrode configuration, with a carbon counter and working electrode and an Ag/AgCl reference electrode. The reference electrode potential was stabilized by applying an Ag/AgCl paste on the carbon ink before the measurements. For each experiment, magnetic beads were suspended in buffer, separated magnetically, and resuspended in 50 μL of 1 mM ferricyanide in 0.1 M KCl. This suspension was then deposited onto the screen-printed working electrode.

EIS measurements were acquired by applying a 5 mV AC perturbation over a frequency range of 1 Hz to 1 MHz, with a 2 s quiet time between data points. Impedance data were analyzed and fitted using CHI software (Version 21.04) with an equivalent circuit model. All parameters and measurement settings were kept constant to assess the influence of magnetic bead size on electrochemical behavior.

### 2.6. Absorbance Measurements Following Conjugation

UV-Vis spectroscopy was used to evaluate the conjugation of AgNPs to magnetic beads of varying sizes. Spectra were recorded over a wavelength range of 190–800 nm with 1 nm resolution. Measurements were performed on both the AgNP-MB conjugates and the supernatants collected after magnetic separation to assess the presence of unbound AgNPs or DNA. The absorbance spectra of the conjugates and supernatants were compared to confirm successful conjugation and to detect changes in characteristic plasmon resonance peaks. Variations in absorbance intensity at specific wavelengths were used as indicators of conjugation efficiency.

### 2.7. SEM Images of Conjugates

SEM was employed to visually and morphologically confirm the conjugation of AgNPs to magnetic beads. Samples were prepared by depositing AgNP-DNA-magnetic bead conjugates onto Au deposited carbon screen printed electrodes and allowing them to dry under ambient conditions. Imaging was performed using a dual-beam focused ion beam system under conditions optimized for high-resolution surface characterization. SEM micrographs were examined to assess the distribution MB-AgNP conjugates on the electrode and surface coverage of AgNPs on the magnetic bead surfaces, thereby verifying successful conjugation.

## 3. Results and Discussion

Three AgNP diameters (20, 50, and 100 nm) and five MB diameters (100, 200, 1000, 3000, and 4500 nm) were selected for the particle size-comparison studies, covering the typical size range used in biological assays. To systematically examine the effect of AgNP size, 1000 nm MBs—a commonly used size in biosensing—were conjugated with each of the three AgNP sizes. For the MB size dependence study, the AgNP size that yielded the highest detection efficiency was conjugated with MBs of different diameters. The optimum AgNP-MB combination was then determined via Au-Ag galvanic exchange-based electrochemical detection.

The efficiency of the galvanic exchange reaction was assessed in terms of silver collection efficiency (AgCE%), defined as the proportion of silver detected relative to the total silver initially present in the labels. In practice, the Ag-Au galvanic exchange reaction does not achieve complete conversion during analysis, and only a fraction of the total Ag atoms are oxidized to Ag^+^ and subsequently detected. The AgCE% was calculated according to Equation (1):

AgCE% = Q/(c × N × V × F) × 100%(1)

where Q is the integrated Ag oxidation charge (C) from ASV following galvanic exchange, F is the Faraday constant (96,500 C·mol^−1^), c is the concentration of Ag labels (mol·L^−1^), N is the average number of Ag atoms per nanoparticle, and V is the assay sample volume (L).

To enable valid size comparisons, systematic normalization strategies were applied. For AgNP size dependence, both the ASV signal and AgCE% were normalized using two approaches: (i) by maintaining the same total number of AgNPs across assays of different diameters, and (ii) by using the same total number of silver atoms across different AgNP sizes. In practice, this meant comparing the ASV signals generated by equal particle counts of 20, 50, and 100 nm AgNPs, as well as comparing signals when the assays contained equivalent total silver content. The number of silver atoms per nanoparticle was estimated based on a face-centered cubic (FCC) lattice structure for silver.

For MB size dependence, normalization was performed using two complementary strategies: (i) equalizing the total mass of MBs of different diameters and (ii) equalizing the total MB surface area available in the assay. In other words, identical mass quantities (mg) of MBs of varying sizes and equivalent total surface areas of MBs were tested to evaluate their effect on the ASV response.

### 3.1. Detection of Silver Nanoparticles of Varying Sizes via Biotin-Streptavidin and Aptamer-Streptavidin Interactions

Calibration plots were developed by varying AgNP concentration for different particle sizes as an initial step to establish the analytical range of detection. From these data, the limits of detection (LOD) and linear ranges were determined for each AgNP size, which, in turn, allowed for the identification of the appropriate normalization concentration and corresponding Ag atom quantity for size-comparison studies. Both biotin-streptavidin and aptamer-streptavidin interactions were evaluated. To confirm that the aptamer-streptavidin interaction was specific and that no nonspecific binding occurred, control experiments were performed using mismatched surface chemistries (citrate-capped AgNPs with streptavidin-MBs, biotin-AgNPs with carboxyl-MBs, and aptamer-AgNPs with carboxyl-MBs), all of which produced no measurable GE signal (see Figure S2).

**Figure 1:**
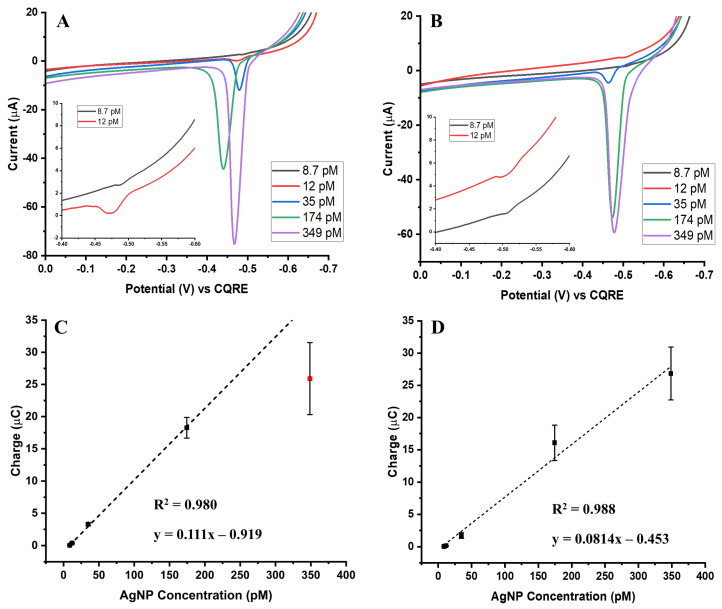
Effect of 20 nm diameter AgNP concentration on electrochemical oxidation signals using different conjugation strategies. ASV overlays for the (**A**) biotin-streptavidin and (**B**) aptamer-streptavidin interactions at increasing AgNP concentrations. Calibration curves of integrated oxidation charge versus AgNP concentration for the (**C**) biotin-streptavidin and (**D**) aptamer-streptavidin systems.

For 20 nm AgNPs, the oxidation signal intensity increased proportionally with AgNP concentration in both interaction types. Figure 1A and Figure 1B present representative ASVs for the biotin-streptavidin and aptamer-streptavidin interactions, respectively. Importantly, the larger 96-base aptamer produced a signal comparable to that of the shorter 12-base biotin linker, demonstrating that the increased number of nitrogenous bases did not introduce significant steric hindrance or compromise electrochemical performance.

To assess the quantitative relationship between AgNP concentration and the corresponding oxidation signal, calibration curves were constructed from the integrated ASV oxidation peaks. Each calibration point represents the mean of five independent measurements performed on screen printed electrodes. Figure 1C and Figure 1D show the resulting calibration curves for biotin-streptavidin and aptamer-streptavidin interactions, respectively.

Despite the known surface inhomogeneity of carbon-based screen printed electrodes, reproducible electrochemical signals were obtained, as reflected by the <20% of relative standard deviations across replicates. Between the two binding strategies, the biotin-streptavidin interaction reached saturation at 174 pM, while the aptamer-streptavidin interaction exhibited an extended linear range up to 349 pM. This suggests that the aptamer-based system provides a more robust quantitative performance across a broader dynamic range.

Moreover, the slopes of the calibration plots reveal important differences in sensitivity. A steeper slope, observed in the biotin-streptavidin system, corresponds to higher sensitivity at lower concentrations. In contrast, the aptamer-streptavidin system, although less steep, maintained linearity over a wider concentration range, making it advantageous for assays requiring broader detection windows.

**Figure 2:**
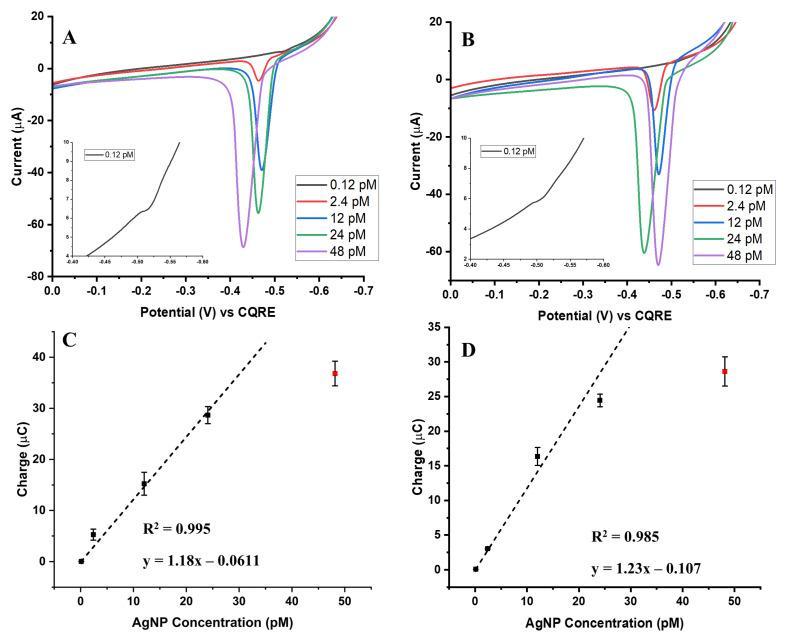
Effect of 50 nm diameter AgNP concentration on electrochemical oxidation signals using different conjugation strategies. ASV overlays for the (**A**) biotin-streptavidin and (**B**) aptamer-streptavidin interactions at increasing AgNP concentrations. Calibration curves of integrated oxidation charge versus AgNP concentration for the (**C**) biotin-streptavidin and (**D**) aptamer-streptavidin systems.

For the 50 nm AgNPs, calibration responses for both the biotin-streptavidin and aptamer-streptavidin conjugates exhibited trends similar to those observed with the 20 nm particles. The overlay ASVs (Figure 2A,B) clearly show that oxidation signal intensity increases with rising nanoparticle concentration for both conjugation strategies. Calibration curves derived from the integrated oxidation charges (Figure 2C,D) demonstrated strong linearity across the tested concentration range, with R^2^ values of 0.995 for the biotin-streptavidin system and 0.985 for the aptamer-streptavidin system. The slopes of these calibration curves were steeper than those obtained with 20 nm AgNPs, reflecting 10–15 times higher sensitivity.

A notable difference compared to the 20 nm system is the faster approach to maximum signal intensity observed with the 50 nm particles. Both conjugation types exhibited an earlier onset of signal saturation, suggesting that larger nanoparticles provide greater initial signal amplification but reach their binding or electrochemical capacity more quickly. Despite this saturation effect, the overall signal strength of the 50 nm AgNPs was more pronounced than that of the 20 nm AgNPs, confirming that nanoparticle size plays a critical role in dictating electrochemical performance. Taken together, these findings indicate that while both particle sizes follow the same general trend of increasing oxidation signal with increasing concentration, the 50 nm AgNPs offer enhanced sensitivity at lower concentrations, but exhibit reduced linear range compared to the smaller 20 nm particles. This balance between sensitivity and dynamic range is important when selecting nanoparticle size for assay optimization.

**Figure 3:**
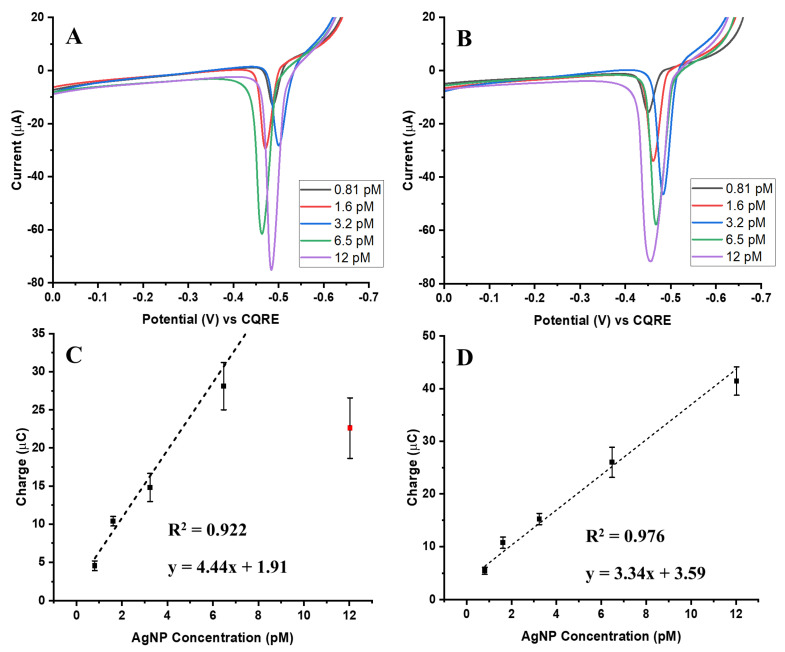
Effect of 100 nm diameter AgNP concentration on electrochemical oxidation signals using different conjugation strategies. ASV overlays for the (**A**) biotin-streptavidin and (**B**) aptamer-streptavidin interactions at increasing AgNP concentrations. Calibration curves of integrated oxidation charge versus AgNP concentration for the (**C**) biotin-streptavidin and (**D**) aptamer-streptavidin systems.

For the 100 nm AgNPs, the overall behavior mirrored that observed for the smaller nanoparticles, with oxidation signal increasing as AgNP concentration increased for both the biotin-streptavidin and aptamer-streptavidin conjugates. The overlay ASVs in Figure 3A,B show this clear concentration-dependent response for both conjugation strategies. Calibration curves derived from the integrated oxidation charges (Figure 3C,D) confirmed strong concentration-dependent responses. As with the 20 nm and 50 nm AgNPs, the aptamer-streptavidin system provided a more consistent linear response across the tested range, whereas the biotin-streptavidin system reached its saturation point earlier.

Importantly, the larger 100 nm AgNPs reached signal saturation more rapidly than the smaller 20 nm and 50 nm particles. This is reflected in the calibration slopes, which increased with particle size: approximately 0.1 µA/pM for 20 nm AgNPs, 1.2 µA/pM for 50 nm AgNPs, and 3.3–4.4 µA/pM for 100 nm AgNPs across both assay types. This behavior is explained by the greater silver mass contained within each individual nanoparticle: a single 100 nm particle contributes far more Ag atoms than a 20 nm or 50 nm particle. Consequently, the apparent signal increase is not solely due to the number of particles, but also reflects the total Ag mass delivered per binding event.

This observation emphasizes the need to evaluate AgCE% rather than relying only on integrated charge. Since galvanic exchange only oxidizes a fraction of the silver present in each label, AgCE% provides a more accurate measure of the detection efficiency by accounting for the actual fraction of silver atoms detected relative to the total available. In this context, charge alone can exaggerate the apparent performance of larger AgNPs, whereas AgCE% normalizes the response to reveal the true effectiveness of the galvanic exchange-based detection strategy.

Overall, while the 100 nm AgNPs produced the strongest oxidation signals, their rapid saturation and high per-particle silver content highlight the importance of AgCE% as the critical parameter for meaningful size-dependent comparisons, which will be further discussed in the next sections.

### 3.2. Effect of AgNP Diameter on GE Signal: Comparison with the Same Number of AgNPs per Asay

To directly compare electrochemical responses based on particle size, assays were normalized to the same number of AgNPs, ensuring that 20, 50, and 100 nm particles were present in equal counts. Based on the previously discussed calibration plots, a concentration of 12 pM was selected for all three sizes to eliminate variability in particle number and isolate the influence of nanoparticle size on the electrochemical signal.

**Figure 4:**
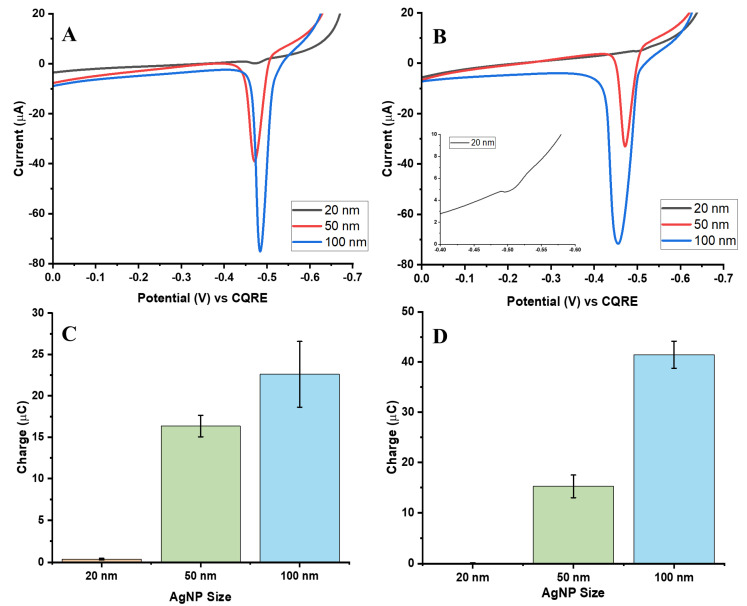
Comparison of GE signals for different AgNP sizes normalized to the same particle count per assay (12 pM). ASV overlays for the (**A**) biotin-streptavidin and (**B**) aptamer-streptavidin interactions. Bar charts of integrated oxidation charge for (**C**) biotin-streptavidin and (**D**) aptamer-streptavidin interactions.

Figure 4A and Figure 4B present the ASVs for the biotin-streptavidin and aptamer-streptavidin interactions after particle count normalization. As expected, larger AgNPs (50 and 100 nm) produced higher oxidation signals than 20 nm particles, indicating that increased particle size enhances the electrochemical response for the same number of nanoparticles. This effect arises from the greater surface area and higher silver content per particle, both of which facilitate more efficient electron transfer and promote the galvanic exchange process [20]. Figure 4C,D show bar charts of charge distribution for each system, highlighting reproducibility and variability across five independent trials.

To assess collection efficiency, the measured charge was compared with the theoretical charge expected from the total number of Ag atoms per assay. Detailed AgCE% values are summarized in Table 1 for both interaction types. The results reveal that 50 nm AgNPs achieved the highest AgCE%, while 100 nm particles showed reduced efficiency, particularly in the aptamer-streptavidin system. This suggests that 50 nm particles provide an optimal balance between sufficient surface area and efficient GE, whereas the larger 100 nm particles, despite their higher total Ag mass, undergo incomplete exchange and saturate more rapidly.

These findings align with previous reports that intermediate nanoparticle sizes maximize both conjugation efficiency and electrochemical performance [10]. Consistently, the 50 nm AgNPs yielded the lowest LOD value for both conjugation strategies, reinforcing their suitability as the optimal size for GE-based detection in this assay format. The LOD was calculated using the following equation: LOD = 3 s/m, where s represents the standard deviation of the response at the lowest measurable concentration, and m is the slope of the corresponding calibration plot.

### 3.3. Effect of AgNP Diameter on GE Signal: Comparison with the Same Number of Ag Atoms per Assay

To compare electrochemical signals based on the same number of silver atoms per assay, the AgNP concentrations were adjusted so that the total Ag atom content remained constant across the three particle sizes. The following concentrations were used: 174 pM for 20 nm AgNPs, 12 pM for 50 nm AgNPs, and 1.6 pM for 100 nm AgNPs. This normalization ensured that differences in the observed electrochemical signals reflected intrinsic particle properties such as size, rather than variations in total available silver content in the assay.

**Figure 5:**
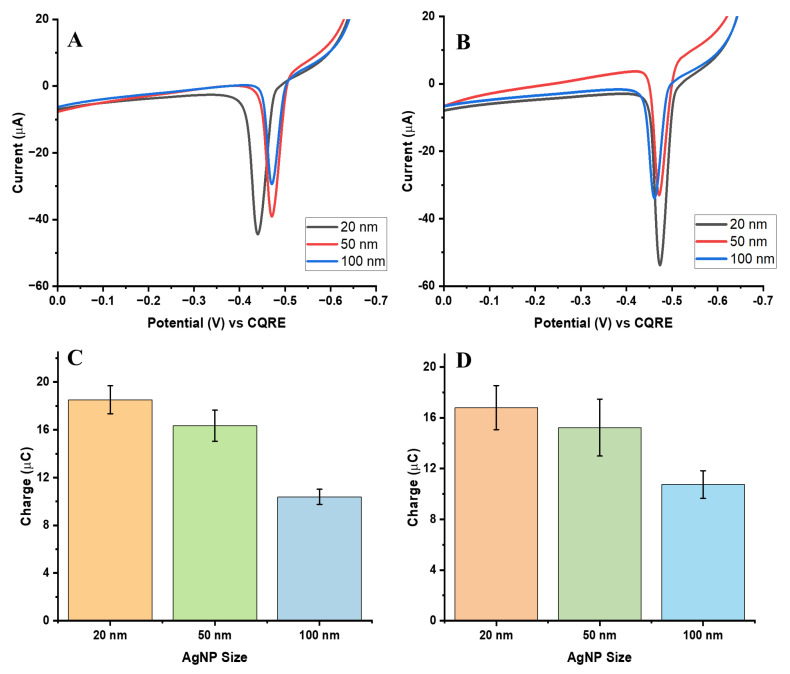
Comparison of GE signals for different AgNP sizes normalized to the same Ag atom count per assay. ASV overlays for the (**A**) biotin-streptavidin and (**B**) aptamer-streptavidin interactions. Bar charts of integrated oxidation charge for (**C**) biotin-streptavidin and (**D**) aptamer-streptavidin interactions.

Figure 5A,B present the AgNP ASVs for the biotin-streptavidin and aptamer-streptavidin interactions under this normalization. As expected, the 20 nm AgNPs produced the highest oxidation signals compared with the larger particles, likely due to their greater surface area-to-volume ratio at the same total number of Ag atoms [19]. Figure 5C,D provide bar charts of collected charge for each particle size, illustrating the distribution and reproducibility of signals across multiple trials.

Although the 20 nm AgNPs generated the largest oxidation signals, this was achieved by using much higher particle concentrations to match the total Ag atom count, which may limit their efficiency in practical assays. In contrast, the 50 nm AgNPs offered the most balanced performance, combining strong electrochemical responses with comparable collection efficiency. The 100 nm AgNPs, while containing more silver mass per particle, showed lower collection efficiency due to incomplete GE and faster saturation (See Table 1).

Overall, the 50 nm AgNPs provided the most favorable GE signals. They not only demonstrated the best charge collection but also yielded the lowest LOD across both normalization approaches—by particle count and by Ag atom count. This outcome makes 50 nm AgNPs the optimal choice among tested sizes for GE-based detection assays, particularly for biosensing applications where low detection limits are critical.

### 3.4. Effect of MB Diameter on GE Signal: Comparison with the Same Mass of MB per Assay

**Figure 6:**
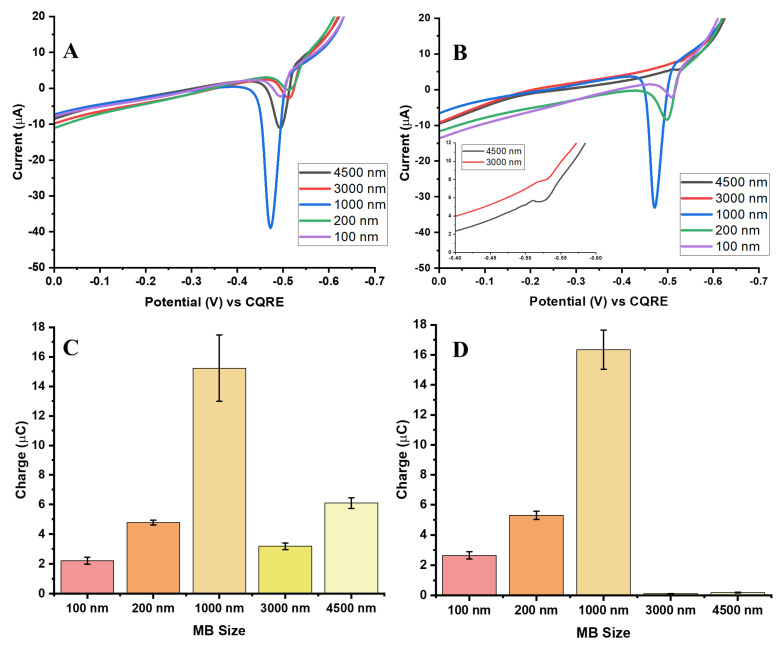
Comparison of GE signals for different MB sizes normalized to the same MB mass per assay. ASV overlays for the (**A**) biotin-streptavidin and **(B**) aptamer-streptavidin interactions. Bar charts of integrated oxidation charge for (**C**) biotin-streptavidin and (**D**) aptamer-streptavidin interactions.

The effect of MB size on Ag-Au GE detection was investigated by varying MB diameters while using a fixed concentration (12 pM) of the previously optimized 50 nm AgNPs. When tested by equal mass (0.02 mg) of MB per assay, larger beads (3000 nm and 4500 nm) exhibited significantly lower Ag oxidation signals than the 1000 nm beads, indicating that oversized beads hinder efficient charge transfer. The diminished response is most likely due to physical obstruction of the electrode surface and reduced accessibility of electro-generated Au^3+^ ions to AgNP labels, thereby lowering overall GE efficiency. Figure 6A,B show representative AgNP ASVs for the biotin-streptavidin and aptamer-streptavidin interactions across bead sizes, highlighting the pronounced dependence of oxidation signal intensity on MB diameter.

Clear differences emerged between the two conjugation strategies. For biotin-streptavidin conjugates, the 3000 nm and 4500 nm diameter bead sizes still generated relatively strong charges (Figure 6C,D), suggesting that biotin linkage supports more effective nanoparticle loading even on large beads. This likely reflects the high affinity and compact geometry of biotin-streptavidin binding, which enables greater AgNP coverage and stronger GE despite bead size. In contrast, the aptamer-streptavidin conjugates produced minimal charge for 3000 nm and 4500 nm beads. This sharp decline is likely due to less efficient aptamer-mediated conjugation and non-uniform AgNP distribution, which limits nanoparticle accessibility for GE. The longer aptamer strands may also increase steric hindrance, reducing the effective packing of AgNPs on larger beads. Moreover, the flexible secondary structures of the long aptamer chains can introduce spatial variability in binding orientation, further decreasing the density of AgNP attachment and weakening the overall electrochemical response.

Smaller beads (100 and 200 nm) also yielded weaker signals than the 1000 nm beads, but still outperformed the largest beads. Their lower responses are attributed to reduced surface area for AgNP conjugation. However, their smaller size improves electrode access for ions allowing for more effective GE than that achieved with larger beads. The thickness of the Au^3+^ diffusion layer provides further insight: MBs larger than this dimension (3000–4500 nm) can physically block ion access to the electrode, thereby restricting charge transfer [20]. By contrast, 1000 nm beads showed optimal size to provide substantial surface area for conjugation, but not so large MBs that they hinder diffusion or block the electrode. Consequently, the 1000 nm MBs consistently delivered the strongest and most efficient electrochemical GE responses across both conjugation strategies.

### 3.5. Effect of MB Diameter on GE Signal: Comparison with the Same Surface Area of MB per Assay

**Figure 7:**
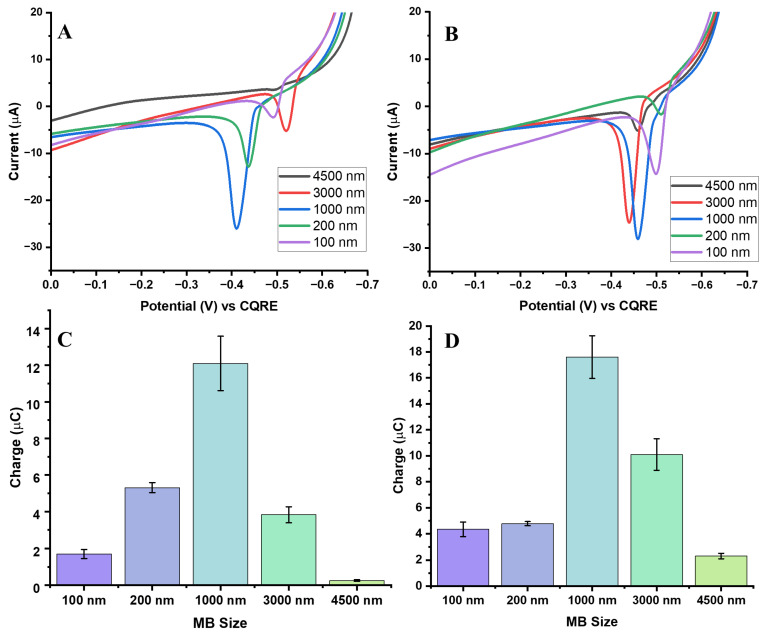
Comparison of GE signals for different MB sizes normalized to the same MB surface area per assay. ASV overlays for the (**A**) biotin-streptavidin and (**B**) aptamer-streptavidin interactions. Bar charts of integrated oxidation charge for (**C**) biotin-streptavidin and (**D**) aptamer-streptavidin interactions.

In this part of this study, the GE signal was normalized based on the estimated total surface area of the MBs (6.9 × 10^14^ nm^2^) used in each assay. This approach was chosen to better reflect the effective interaction area available for GE with Au^3+^ ions, ensuring that observed differences in signal were due to bead size and geometry rather than bead mass alone. Figure 7A and Figure 7B show representative AgNP ASVs for biotin-streptavidin and aptamer-streptavidin interactions, respectively, across MB sizes.

When normalized by surface area, the 1000 nm MBs continued to yield the strongest Ag oxidation signals, consistent with the mass-normalized results. However, surface area normalization revealed higher signals for the 3000 nm and 4500 nm beads than in the mass-normalization approach, suggesting that their larger surface area partly offsets the steric and diffusion limitations associated with their size. In contrast, the 100 nm and 200 nm beads produced weaker signals, as expected from their smaller conjugation surface per binding event, though their responses were still greater than those of the largest beads.

Figure 7C,D display the charge distributions for the different MB sizes after surface area normalization. These results confirm that the 1000 nm beads consistently produced the most robust and reproducible signals, while the larger beads (3000 and 4500 nm) showed improved charge collection compared to mass normalization but remained less efficient overall. This reduced efficiency is likely due to their physical obstruction of the electrode surface, which hinders charge transfer despite their larger available surface area.

The comparison between mass and surface area normalization highlights complementary aspects of MB behavior. Mass normalization controls for the total amount of bead material, thereby showing how bulk quantities influence signal. However, this approach underestimates the contribution of bead surface availability. Surface area normalization, in contrast, provided a more accurate representation of the effective bead-electrode interaction, particularly for larger beads where accessible surface area becomes the key factor. Even under this normalization, the 1000 nm MBs clearly outperformed both smaller and larger beads, demonstrating the best representation of surface area availability and Au^3+^ ion accessibility thus efficient GE.

It is noteworthy that small shifts in the ASV peak potentials were observed across measurements, primarily due to the use of a carbon quasi-reference electrode, which can exhibit minor potential drift compared to a stable Ag/AgCl reference. These variations may also arise from slight differences in galvanic exchange kinetics and local electrode surface heterogeneity inherent to screen-printed electrodes.

### 3.6. Electrochemical Impedance Spectroscopy Study of Varying Magnetic Bead Sizes on Carbon Screen-Printed Electrodes

Our results clearly showed that 1000 nm diameter beads exhibit superior GE behavior when normalized by both bead mass and bead surface area. Specifically, 1000 nm beads demonstrated approximately two-fold higher AgCE% compared to both smaller and larger beads. This indicates that the 1000 nm beads allow for more effective access to diffusing Ag and Au ions during the GE process.

**Scheme 2:**
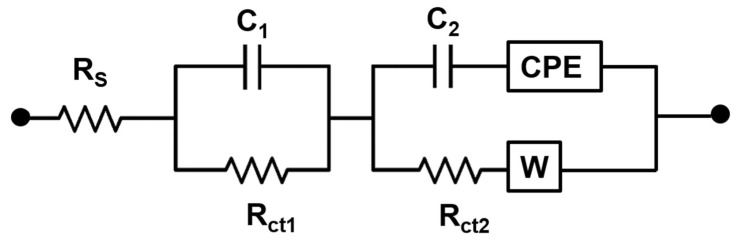
Equivalent circuit model used to fit Nyquist plots obtained from EIS measurements.

To further evaluate this, we investigated the charge transfer impedance generated on carbon screen printed electrodes when different bead sizes were deposited. The EIS data were analyzed by fitting into an equivalent circuit model, as shown in Scheme 2.

This circuit comprised solution resistance (R_s_), two charge transfer resistances (R_ct1_ and R_ct2_), two double-layer capacitances (C1 and C2), Warburg impedance (W) and a constant phase element (CPE). The R_s_ represents the resistance of the electrolyte and is placed in series with the rest of the circuit components. The R_ct_ elements reflect the resistance to electron transfer at the electrode surface, and are modeled in parallel double-layer capacitors. The Warburg impedance is placed in parallel to model diffusion processes at the electrode surface, particularly at low frequencies where ion transport becomes the dominant factor. A constant-phase element is incorporated into the equivalent circuit to account for non-ideal capacitive behavior at the electrode-electrolyte interface, arising from surface roughness, inhomogeneous current distribution, and the heterogeneous nature of the carbon screen-printed electrode modified with magnetic beads [28,29].

**Figure 8:**
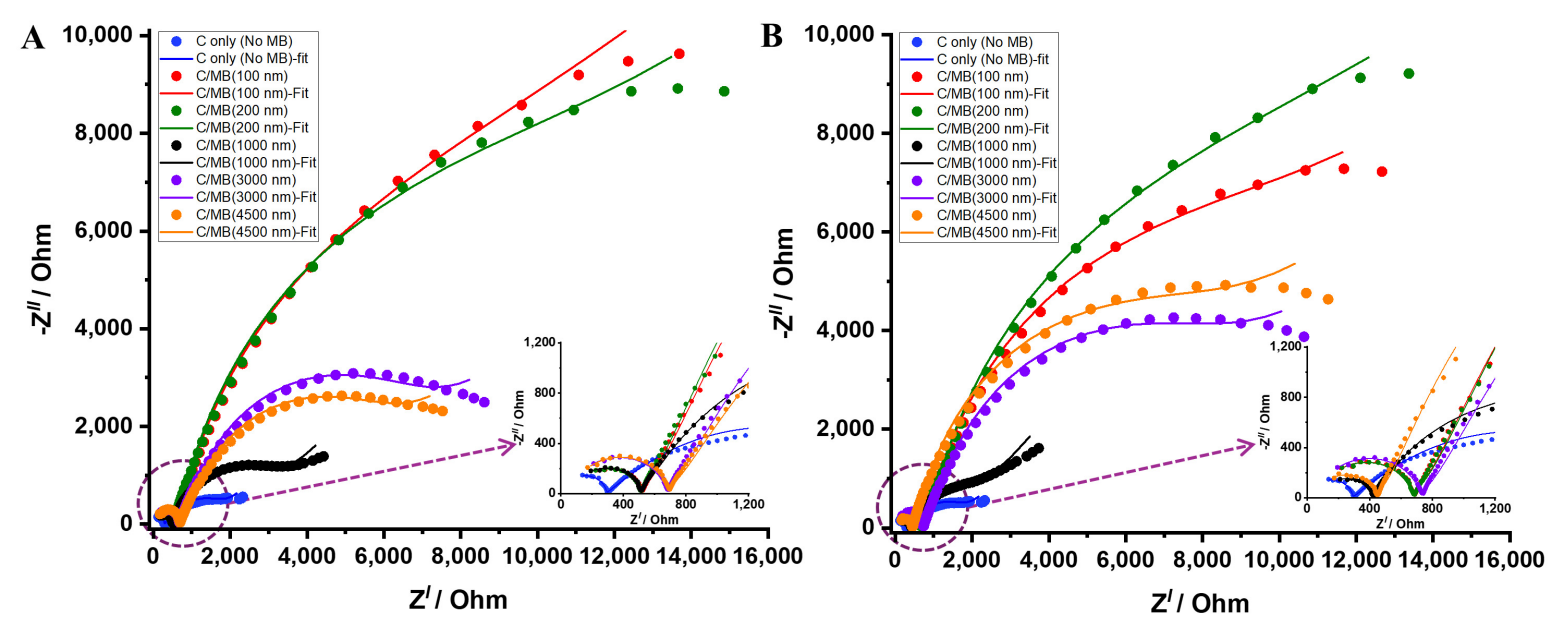
Nyquist plots for magnetic bead size dependence using a 1 mM ferricyanide (FCN) redox probe in 0.1 M KCl electrolyte. (**A**) Normalization by same mass of magnetic beads per assay. (**B**) Normalization by same total surface area of magnetic beads per assay. Experimental impedance data are shown as solid circles, while solid lines represent the fitted curves based on the equivalent circuit model. Insects display the zoomed-in low-impedance regions for clarity.

The Nyquist plots for different bead sizes under both normalization strategies are shown in Figure 8. These EIS results reveal two distinct charge transfer resistance components (R_ct1_ and R_ct2_), corresponding to two separate time constants observed in the Nyquist plots [30]. The first semicircle, associated with R_ct1_ values ranging from ~300–600 Ω (Table S1), represents the faster interfacial charge-transfer process occurring at the electrode-electrolyte interface. The second semicircle R_ct2_, with much higher values, reflects the slower charge-transfer process across the magnetic bead layer, where the presence of insulating MBs introduces additional diffusion and electron-transfer barriers. Under mass normalization (Figure 8A), smaller beads (100 and 200 nm) exhibited higher impedance than any other bead size. Their small size led to dense packing at the electrode surface, obstructing electrode access and limiting the ability of the ferricyanide probe to interact with the electrode. This arrangement of MB may have resulted in higher R_ct2_ values, as summarized in Table S1. In contrast, the 1000 nm beads showed markedly lower R_ct2_ value, suggesting reduced surface obstruction and an optimal balance between surface coverage and accessibility. Despite being larger, these beads covered less of the electrode area, allowing more efficient charge transfer and lower impedance. Moreover, the 3000 nm and 4500 nm beads again exhibited high R_ct2_ values—though lower than the smaller beads—indicating that excessive bead size also hampers electrochemical efficiency through steric obstruction and limited contact with the electrode. These results imply that both excessively small and large MBs weaken electron transfer, while intermediate sizes promote optimal charge transport at the interface [24].

Surface area normalization (Figure 8B) provided additional insight by equalizing the effective bead surface area across assays, allowing for a direct comparison of how surface availability—rather than total bead mass—affects GE behavior. Under these conditions, the 1000 nm beads again exhibited the lowest R_ct2_ values (Table S1), confirming their superior charge-transfer efficiency. Interestingly, the smaller beads (100 and 200 nm) produced higher impedance than the larger beads (3000 and 4500 nm), suggesting that smaller beads offer higher bead density, which restricts efficient charge transfer. Conversely, the larger beads, though fewer in number, provide broader contact areas but still introduce steric obstruction and hinder ion diffusion near the electrode surface. Overall, both extremes showed higher R_ct2_ values than the 1000 nm beads, reaffirming that this intermediate size offers the most favorable balance between surface accessibility and electrochemical efficiency for effective galvanic exchange [23].

### 3.7. Analysis of Conjugation Efficiency via UV/Vis Absorbance Measurements

UV/Vis absorbance was used to evaluate the efficiency of AgNP conjugation to MBs for both biotin-streptavidin and aptamer-streptavidin interactions (Figure S3). The AgNP-DNA-biotin or AgNP-Aptamer spectra served as references, with the surface plasmon resonance peak at ~416 nm corresponding to unconjugated silver. Higher absorbance after conjugation indicates more free AgNPs in solution, whereas lower absorbance reflects greater conjugation to MBs [13,22].

For both conjugation strategies, the 100 nm and 200 nm magnetic beads exhibited relatively high absorbance values, indicating incomplete conjugation and a greater amount of unbound silver remaining in solution. This observation is likely due to their lower magnetic content, which reduces capture efficiency during magnetic separation, as well as increased light scattering from smaller particles. In contrast, the 3000 nm and 4500 nm beads in the aptamer-streptavidin system also displayed higher absorbance despite their larger surface area. This behavior may result from steric hindrance, electrostatic repulsion, or bead aggregation that limits AgNP attachment. Additionally, the aptamer-streptavidin conjugation occurs via a 96-base aptamer sequence compared to the 12-base biotin-streptavidin linkage, which can introduce variations in binding orientation and spatial arrangement, further influencing conjugation efficiency.

In both interaction types, 1000 nm MBs consistently produced the lowest absorbance, indicating the most efficient AgNP conjugation. These beads appear to provide the best balance of surface area, accessibility, and minimal obstruction, resulting in reduced free AgNPs and more effective binding compared to smaller or larger beads.

### 3.8. SEM Characterization of Surface Morphology

SEM was employed to examine both the bare electrode surface and the conjugation of AgNPs to MBs. Figure S4A shows the surface of the Au deposited carbon screen-printed electrode, which displays notable roughness and inhomogeneity. These surface features are important for interpreting the EIS data, as they can influence charge transfer, electric field distribution, and ion diffusion at the interface. The observed non-idealities provide justification for employing a more complex equivalent circuit, where two parallel capacitances and charge transfer resistance elements were included to account for heterogeneous surface behavior [31].

To confirm successful conjugation, SEM imaging was also conducted on AgNP-MB complexes. As shown in Figure S4B, silver nanoparticles are clearly attached to the MB surface, demonstrating that the conjugation process proceeded effectively. The uniform attachment of AgNPs provides direct visual confirmation of efficient binding, consistent with the EIS results and UV/Vis absorbance measurements. In particular, the uniform distribution observed for the 1000 nm beads aligns with their superior performance, as reflected by lower impedance values and stronger conjugation efficiency. These SEM observations therefore corroborate the electrochemical and optical findings, highlighting the effective role of 1000 nm MBs in optimizing assay performance.

## 4. Conclusions

In this study, we systematically evaluated the influence of AgNP size and magnetic bead size on electrochemical assays using biotin-streptavidin and aptamer-streptavidin interactions. Our findings show that 50 nm AgNPs provide the most balanced electrochemical GE response, achieving optimal conjugation efficiency and charge transfer performance. In contrast, 1000 nm magnetic beads also demonstrated the highest silver collection efficiency, reflecting their ideal balance between surface area and mass. Both larger and smaller MB sizes produced higher impedance, likely due to physical obstruction of the electrode surface and limited conjugation efficiency.

UV/Vis absorbance confirmed these trends, with the 1000 nm beads showing the most efficient conjugation, whereas both smaller and larger beads retained some unbound silver. SEM imaging further validated the successful conjugation of AgNPs, with uniform attachment most evident for the 1000 nm beads. Together, these results highlight the strong influence of particle size and bead size on conjugation efficiency and galvanic exchange performance.

Overall, this work provides important design considerations for AgNP-magnetic bead electrochemical assays. The combination of 50 nm AgNPs with 1000 nm magnetic beads emerges as the most effective configuration, offering strong sensitivity, reproducibility, and electrochemical performance. These findings lay the groundwork for future biomarker detection studies, particularly in point-of-care diagnostic applications, where optimized nanoparticle-bead systems could enable highly sensitive and reliable biosensing.

## Figures and Tables

**Table 1:** LOD and AgCE% values for biotin-streptavidin and aptamer-streptavidin interactions using AgNPs of different sizes.

Interaction Type	AgNP Size	LOD (pM)	AgCE% When Same Number of AgNPs Used per Assay	AgCE% When Same Number of Ag Atoms Used per Assay
Biotin-Streptavidin	20 nm	0.32	2.7 ± 0.7	8.9 ± 1.3
	50 nm	0.02	7.4 ± 0.6	7.4 ± 0.6
	100 nm	0.42	1.3 ± 0.2	4.3 ± 0.3
Aptamer-Streptavidin	20 nm	0.41	1.1 ± 0.2	7.8 ± 1.3
	50 nm	0.02	6.9 ± 1.0	6.9 ± 1.0
	100 nm	0.60	2.3 ± 0.2	4.5 ± 0.5

## Data Availability

The raw data supporting the conclusions of this article will be made available by the corresponding author on request.

## Supplementary Materials

The following supporting information can be downloaded at: https://www.mdpi.com/article/10.3390/bios15120768/s1, Figure S1 illustrates the fabrication process of the screen-printed electrode; Figure S2 presents control experiments for MB-AgNP conjugate formation; Figure S3 shows UV/Vis characterization of MB-AgNP conjugates; and Figure S4 provides SEM images confirming surface morphology and conjugation. Table S1 summarizes the fitted parameters obtained from electrochemical impedance spectroscopy for electrodes modified with magnetic beads of varying diameters.

## References

[1] GeGen S., Meng G., Aodeng G., Ga L., Ai J. (2025). Advances in aptamer-based electrochemical biosensors for disease diagnosis: Integration of DNA and nanomaterials. Nanoscale Horiz..

[2] Ni S., Zhuo Z., Pan Y., Yu Y., Li F., Liu J., Wang L., Wu X., Li D., Wan Y. (2021). Recent Progress in Aptamer Discoveries and Modifications for Therapeutic Applications. ACS Appl. Mater. Interfaces.

[3] Sefah K., Shangguan D., Xiong X., O’Donoghue M.B., Tan W. (2010). Development of DNA aptamers using Cell-SELEX. Nat. Protoc..

[4] Lam S.Y., Lau H.L., Kwok C.K. (2022). Capture-SELEX: Selection Strategy, Aptamer Identification, and Biosensing Application. Biosensors.

[5] Walgama C., Raj N. (2023). Silver nanoparticles in electrochemical immunosensing and the emergence of silver–gold galvanic exchange detection. Chem. Commun..

[6] Chandrasekar N., Alexander P.S., Pham P.T., Ramachandran B., Nivedha D.R., Shekar N., Muraleetharan M., Kiran S.K., Hwang M.T. (2025). Exploring the Potential of MXenes for Biomedical and Environmental Applications—An Abridged Review. Luminescence.

[7] Lee S., Peng R., Wu C., Li M. (2022). Programmable black phosphorus image sensor for broadband optoelectronic edge computing. Nat. Commun..

[8] Lu S.-M., Peng Y.-Y., Ying Y.-L., Long Y.-T. (2020). Electrochemical Sensing at a Confined Space. Anal. Chem..

[9] Pollok N.E., Rabin C., Smith L., Crooks R.M. (2019). Orientation-Controlled Bioconjugation of Antibodies to Silver Nanoparticles. Bioconjug. Chem..

[10] Kogan M.R., Pollok N.E., Crooks R.M. (2018). Detection of Silver Nanoparticles by Electrochemically Activated Galvanic Exchange. Langmuir.

[11] Cunningham J.C., Kogan M.R., Tsai Y.-J., Luo L., Richards I., Crooks R.M. (2016). Paper-Based Sensor for Electrochemical Detection of Silver Nanoparticle Labels by Galvanic Exchange. ACS Sens..

[12] Pollok N.E., Rabin C., Walgama C.T., Smith L., Richards I., Crooks R.M. (2020). Electrochemical Detection of NT-proBNP Using a Metalloimmunoassay on a Paper Electrode Platform. ACS Sens..

[13] Walgama C., Nguyen M.P., Boatner L.M., Richards I., Crooks R.M. (2020). Hybrid paper and 3D-printed microfluidic device for electrochemical detection of Ag nanoparticle labels. Lab A Chip.

[14] Raj N., Crooks R.M. (2022). Detection Efficiency of Ag Nanoparticle Labels for a Heart Failure Marker Using Linear and Square-Wave Anodic Stripping Voltammetry. Biosensors.

[15] Raj N., Crooks R.M. (2022). Plastic-based lateral flow immunoassay device for electrochemical detection of NT-proBNP. Analyst.

[16] Peng Y., Raj N., Strasser J.W., Crooks R.M. (2022). Paper Biosensor for the Detection of NT-proBNP Using Silver Nanodisks as Electrochemical Labels. Nanomaterials.

[17] Pollok N.E., Peng Y., Raj N., Walgama C., Crooks R.M. (2021). Dual-Shaped Silver Nanoparticle Labels for Electrochemical Detection of Bioassays. ACS Appl. Nano Mater..

[18] Peng Y., Rabin C., Walgama C.T., Pollok N.E., Smith L., Richards I., Crooks R.M. (2021). Silver Nanocubes as Electrochemical Labels for Bioassays. ACS Sens..

[19] Ivanova O.S., Zamborini F.P. (2009). Size-Dependent Electrochemical Oxidation of Silver Nanoparticles. J. Am. Chem. Soc..

[20] Pattadar D.K., Masitas R.A., Stachurski C.D., Cliffel D.E., Zamborini F.P. (2020). Reversing the Thermodynamics of Galvanic Replacement Reactions by Decreasing the Size of Gold Nanoparticles. J. Am. Chem. Soc..

[21] Perevezentseva D.O., Gorchakov E.V., Vaytulevich E.A. (2023). Features of Silver-Nanoparticle-Based Electrochemical Sensors. Shape and Size Effects. Nanobiotechnol. Rep..

[22] Singh S., Bharti A., Meena V.K. (2015). Green synthesis of multi-shaped silver nanoparticles: Optical, morphological and antibacterial properties. J. Mater. Sci. Mater. Electron..

[23] Hsu W., Shih Y.-T., Lee M.-S., Huang H.-Y., Wu W.-N. (2022). Bead Number Effect in a Magnetic-Beads-Based Digital Microfluidic Immunoassay. Biosensors.

[24] Li F., Xu H., Sun P., Hu Z., Aguilar Z.P. (2019). Size effects of magnetic beads in circulating tumour cells magnetic capture based on streptavidin–biotin complexation. IET Nanobiotechnol..

[25] Wang C., Yang G., Luo Z., Ding H. (2009). In vitro selection of high-affinity DNA aptamers for streptavidin. Acta Biochim. Biophys. Sin..

[26] Stoltenburg R., Reinemann C., Strehlitz B. (2005). FluMag-SELEX as an advantageous method for DNA aptamer selection. Anal. Bioanal. Chem..

[27] Zhang X., Servos M.R., Liu J. (2012). Fast pH-assisted functionalization of silver nanoparticles with monothiolated DNA. Chem. Commun..

[28] Lazanas A.C., Prodromidis M.I. (2023). Electrochemical Impedance Spectroscopy─A Tutorial. ACS Meas. Sci. Au.

[29] Vivier V., Orazem M.E. (2022). Impedance Analysis of Electrochemical Systems. Chem. Rev..

[30] Magar H.S., Hassan R.Y.A., Mulchandani A. (2021). Electrochemical Impedance Spectroscopy (EIS): Principles, Construction, and Biosensing Applications. Sensors.

[31] Ivanov R., Czibula C., Teichert C., Bojinov M., Tsakova V. (2021). Carbon screen-printed electrodes for substrate-assisted electroless deposition of palladium. J. Electroanal. Chem..

