# Supplementary material for: Size Effects of Silver Nanoparticles and Magnetic Beads on Silver-Gold Galvanic Exchange in Aptamer-Based Electrochemical Assays
Source: Biosensors (Basel). 2025 Nov 25;15(12):768. doi: 10.3390/bios15120768 (PMC12730487; doi:10.3390/bios15120768)
Supplement: Supplementary file 1 [file biosensors-15-00768-s001.zip › biosensors-3946008-supplementary.pdf]

biosensors MDPI

Supplementary Materials

Size Effects of Silver Nanoparticles and Magnetic Beads on Silver-Gold Galvanic Exchange in Aptamer-Based Electrochemical Assays

Eman Alwarsh, Trang Bui, Marco Cardenas, Daniel Adrian and Charuksha Walgama *

Department of Mathematical, Applied, and Physical Sciences, University of Houston-Clear Lake, 2700 Bay Area Boulevard, Houston, TX 77058, USA
Correspondence: walgama@uhcl.edu

Table of Contents

Item	Description
Figure S1	Fabrication of the screen-printed electrode
Figure S2	Control Experiments for MB-AgNP conjugate formation
Figure S3	UV/Vis Characterization of MB-AgNP conjugates
Figure S4	SEM Characterization of MB-AgNP conjugates
Table S1	Fitting parameters of electrochemical impedance data

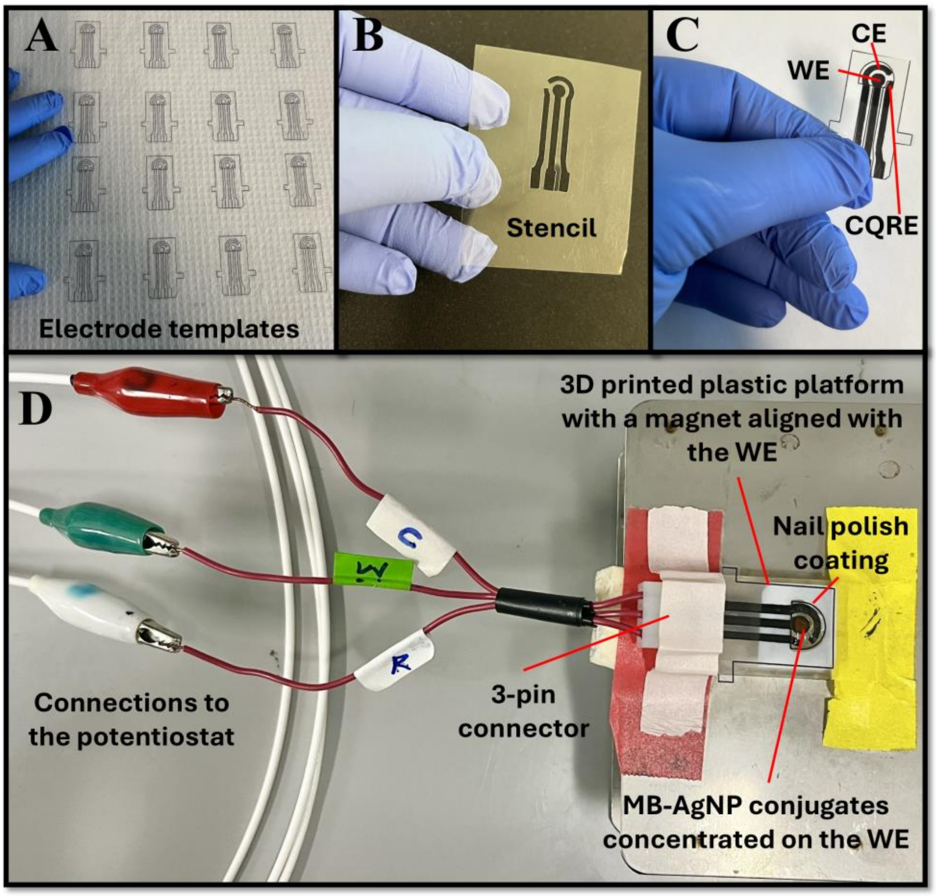

Figure S1. Photographs illustrating the fabrication process of the screen-printed electrode. (A) Printed electrode design templates. (B) Stencil patterned using a Cricut Maker 3 cutting machine. (C) Electrode strip after screen printing with conductive carbon paste (CE = carbon counter electrode, WE (C/Au) = carbon working electrode with electrodeposited Au, and CQRE = carbon quasi-reference electrode). (D) Complete electrode assembly connected to a 3-pin connector for stable ohmic contact with the potentiostat. A 3D-printed plastic extension aligns a magnet beneath the working electrode to concentrate the MB–AgNP conjugates during analysis.

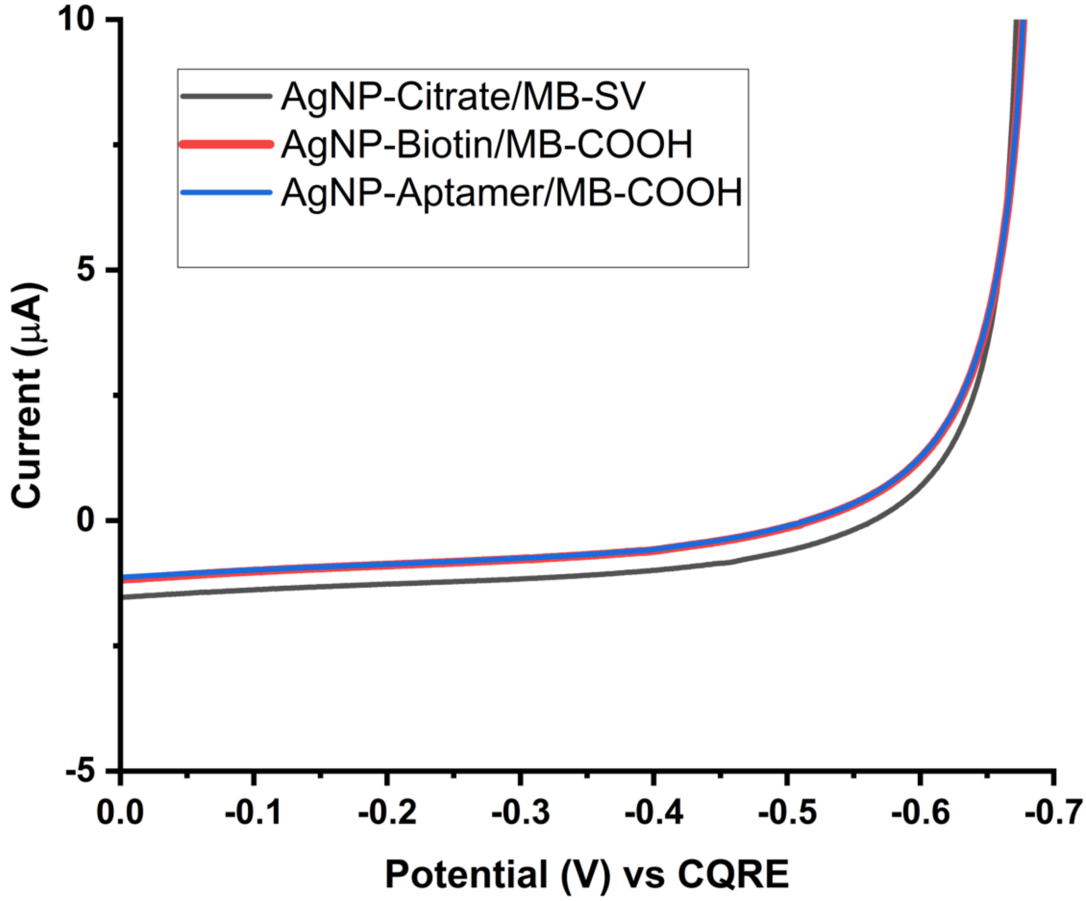

Figure S2. Control GE experiments demonstrating assay specificity. Conjugates prepared using citrate-AgNPs with streptavidin-MBs(black), biotin-AgNPs with carboxyl-MBs (red), and aptamer-AgNPs with carboxyl-MBs (blue) generated no GE signal, confirming the absence of nonspecific interactions.

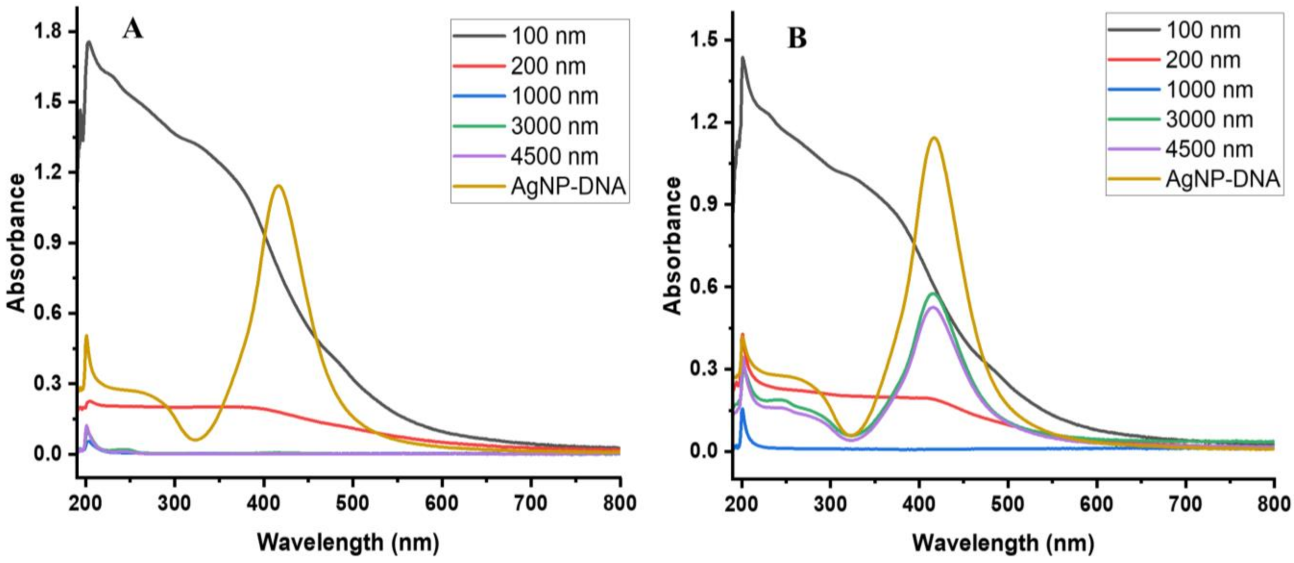

Figure S3. UV/Vis absorbance spectra of the supernatant after AgNP conjugation to MBs of varying diameters. (A) Biotin–streptavidin interaction. (B) Aptamer–streptavidin interaction. Lower absorbance at ~416 nm indicates more efficient AgNP conjugation.

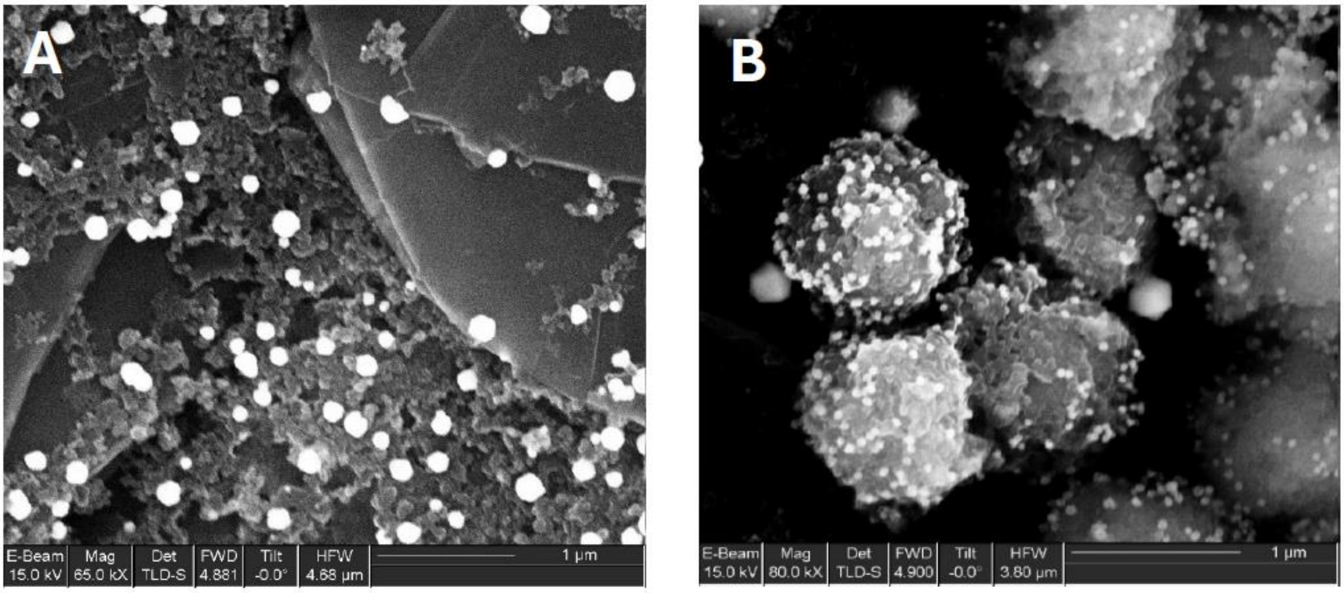

Figure S4. SEM images of deposits on carbon-based SPEs. (A) Au-modified SPE surface. (B) 50 nm AgNP-modified MBs deposited onto Au-modified SPE surface.

Circuit Element	MB Content Normalization by Mass						MB Content Normalization by Surface Area					
	No MB	100 nm	200 nm	1000 nm	3000 nm	4500 nm	No MB	100 nm	200 nm	1000 nm	3000 nm	4500 nm
R_s(ohm)	12	93	94	104	83	84	12	97	92	87	80	87
R_{ct1}(ohm)	287	560	557	314	616	389	287	393	391	401	569	570

R_{ct2}(ohm)	1464	9787	11000	1618	7653	7767	1464	9896	10840	2483	6121	5288
C_1(F)	5.6×10^{-10}	4.8×10^{-10}	4.8×10^{-10}	6.5×10^{-10}	5.0×10^{-10}	6.5×10^{-10}	5.6×10^{-10}	5.3×10^{-10}	5.3×10^{-10}	6.1×10^{-10}	4.8×10^{-10}	6.1×10^{-10}
C_2(F)	7.3×10^{-5}	3.8×10^{-6}	4.0×10^{-6}	2.5×10^{-6}	2.8×10^{-6}	2.7×10^{-6}	7.3×10^{-6}	3.6×10^{-6}	3.0×10^{-6}	2.6×10^{-6}	2.6×10^{-6}	3.2×10^{-6}
W(S.s$^{1/2}$)	1.7×10^{-3}	5.5×10^{-5}	4.3×10^{-5}	1.6×10^{-4}	9.3×10^{-5}	7.2×10^{-5}	1.7×10^{-3}	3.7×10^{-5}	4.1×10^{-5}	2.0×10^{-4}	1.3×10^{-4}	1.5×10^{-4}
CPE(S.s^n)	2.2×10^{-4}	9.7×10^{-5}	9.2×10^{-5}	1.2×10^{-4}	7.6×10^{-5}	1.0×10^{-4}	2.2×10^{-4}	9.5×10^{-5}	9.1×10^{-5}	9.3×10^{-5}	7.7×10^{-5}	7.7×10^{-5}

Table S1. Fitted parameters obtained from electrochemical impedance spectroscopy data for screen-printed electrodes modified with magnetic beads of varying diameters. All parameters fitted <3% error.

Abbreviations:

R_s – solution resistance;

R_{ct_1} – first charge-transfer resistance corresponding to the electrode–electrolyte interface;

R_{ct_2} – second charge-transfer resistance associated with screen-printed carbon surface heterogeneity and the magnetic bead layer;

C_1 and C_2 – double-layer capacitances representing different interfacial processes;

W – Warburg element corresponding to ion-diffusion effects;

CPE – constant phase element used to model non-ideal capacitive behavior due to surface roughness and heterogeneity.
